# Advancing neutron diffraction for accurate structural measurement of light elements at megabar pressures

**DOI:** 10.1038/s41598-023-31295-3

**Published:** 2023-03-23

**Authors:** Bianca Haberl, Malcolm Guthrie, Reinhard Boehler

**Affiliations:** Neutron Scattering Division, Neutron Sciences Directorate, Oak Ridge National Laboratory, Oak Ridge, TN 37830 USA

**Keywords:** Condensed-matter physics, Techniques and instrumentation, Applied physics, Condensed-matter physics, Techniques and instrumentation

## Abstract

Over the last 60 years, the diamond anvil cell (DAC) has emerged as the tool of choice in high pressure science because materials can be studied at megabar pressures using X-ray and spectroscopic probes. In contrast, the pressure range for neutron diffraction has been limited due to low neutron flux even at the strongest sources and the resulting large sample sizes. Here, we introduce a neutron DAC that enables break-out of the previously limited pressure range. Key elements are ball-bearing guides for improved mechanical stability, gem-quality synthetic diamonds with novel anvil support and improved in-seat collimation. We demonstrate a pressure record of 1.15 Mbar and crystallographic analysis at 1 Mbar on the example of nickel. Additionally, insights into the phase behavior of graphite to 0.5 Mbar are described. These technical and analytical developments will further allow structural studies on low-Z materials that are difficult to characterize by X-rays.

It has been over 40 years since the ‘sound barrier’ of achieving the pressure of one megabar (= 100 GPa) was broken in a diamond anvil cell^1^ and the field of high pressure research has dramatically advanced since. Conditions of the Earth’s core and lower mantle can now be simulated and many geophysical questions have been addressed^2^. Similarly, physical understanding of phase diagrams has immensely advanced and, for example, several new high pressure phases were identified in the ‘simplest’ of all materials, hydrogen (see recent review^3^). Furthermore, new materials can now be synthesized through high pressure (and high temperature conditions) such as various nitrides^4–6^ and, as of particular recent interest, superconducting superhydrides, for example^7–9^. Finally, the field continues to be highly active and multi-megabar pressures were recently achieved with sophisticated toroidal-shaped diamond anvils or double-stage techniques^10–12^.

In common to many of these studies is the fact that in situ structure determination under pressure is performed through X-ray diffraction. While such in situ X-ray diffraction is very powerful, there are severe limitations when it comes to low-Z elements. Here, neutron diffraction has evolved as an important tool. Not only are neutrons sensitive to many low-Z elements, neutrons are also capable of distinguishing between different isotopes. As neutrons carry a magnetic moment, they also allow for the detection of magnetic Bragg diffraction. Thus, a number of very important questions in high pressure science can only be addressed by neutron diffraction. For example, for geophysics neutron diffraction can investigate the nature of water within minerals or can provide knowledge of the density and structure of the ices of water, methane, and other light compounds. Neutron diffraction is key for the understanding of phase diagrams of light elements such as hydrogen or carbon. In view of the recently discovered metal superhydrides, neutron diffraction can reveal the exact position of hydrogen in the metal matrix, thus providing important structural information.

However, unlike X-ray diffraction, the relatively much lower neutron flux of existing neutron facilities required relatively large sample volumes thus limiting the pressure to a few tens of GPa. Until recently, typical maximum pressures at most user facilities were limited to $\sim$ 25 GPa when using Paris-Edinburgh cells^13^, although recently breakout studies up to 40 GPa have been reported^14,15^.

To push for higher pressures, several iterations of neutron diamond anvil cells (DAC) have been developed over time. A major body of work commenced at the Kurchatov Institute in Moscow and was later transferred and further improved in France. There, studies up to 40 GPa on materials such as hydrogen^16^ or on magnetic materials have been performed^17^. These studies however, were only able to identify a very small number of reflections, not sufficient for crystallographic analysis and full structure information. Consequently, several efforts world-wide have attempted to further the development of neutron DACs. These efforts focused on high-quality data obtained through single crystal diffraction at the Institute-Laue-Langevin^18,19^ or the Forschungs-Neutronenquelle Heinz Maier–Leibnitz^20^ as well as on higher pressure capabilities using powder diffraction at the Japan Proton Accelerator Research Complex (J-PARC)^21^, the Frank Laboratory of Neutron Physics^22^, as well as at Oak Ridge National Laboratory’s (ORNL) Spallation Neutron Source (SNS)^23,24^.

Reaching megabar pressures in a neutron diffraction experiment is not simply done by up-scaling conventional DACs as used for X-ray diffraction. Typically, for reaching one megabar in a DAC, culet sizes of not more than $\sim$ 100 µm are used. For neutron diffraction, where the sample sizes must be much larger, the minimum culet size has been in the millimeter regime. This larger area increases the force requirement more than 10-fold to several metric tons usually supplied by a gas membrane or hydraulic press. The main problem is that such high forces induce significant shear stresses in the diamond cell body and thus in the diamond anvils and also exceeding the strength of the anvil seats resulting in frequent diamond anvil failure.

Nonetheless, at the SNS efforts have been underway for over 10 years to develop megabar neutron diffraction. These efforts have centered on the high pressure diffractometer Spallation Neutrons and Pressure (SNAP)^25^. SNAP is a high-flux, medium-resolution instrument equipped with advanced neutron focusing optics and large area detectors that uses white-beam time-of-flight (TOF) neutron diffraction. This combination of white-beam TOF diffraction and large angular coverage presents advantages for high pressure studies, particularly increased *Q*-coverage, an ability to evaluate texture as well as an ability to handle blocked or heavily attenuated sections of the 2θ-range (since a significant portion of the full diffraction pattern can be measured at each 2θ due to the energy-dispersive nature of white-beam TOF diffraction). This allows for the use of absorbing materials in the pressure cell set-up, specifically gasket materials, without compromising the *Q*-range, a fact that has highly benefited DAC studies on SNAP^26,27^.

Over time, several iterations of DACs have been developed on SNAP. Initially, these have used highly elaborate anvil support^23^, which successfully achieved over 90 GPa^23^ and obtained high quality crystallographic data on water ice up to 52 GPa^28^. However, the available scattering aperture and thus *Q*-range severely limited the possible studies to simple cubic systems. Even more detrimental, the design was very expensive and not feasible for a user program. The next upgrade thus focused on very large synthetic diamonds grown by chemical vapor deposition (CVD) in order to reduce stresses in the anvil supports^24^. These enabled full crystallographic analysis and structural refinement to 62 GPa^29^ on water ice and were successfully rolled out for a wider user program. More recently, DAC efforts at SNAP have been attempting to take advantage of two key factors, an increased flux of the overall facility (that now operates at 1.4 MW) and significant upgrades to the beamline (an upgraded detector system and a vastly improved neutron focusing guide system). Despite these upgrades however, the maximum pressure in the DACs remained at 65 GPa, a barrier that simply could not be overcome with these large CVD anvils and these cell bodies.

Here, we present the development of novel neutron DACs based on smaller gem-quality synthetic diamond anvils, ultra-high precision cell bodies and new anvil supports as well as high-precision in-built boron collimation for reduced backgrounds. These new DACs take advantage of the recent transformative beamline upgrades, since higher flux allows for smaller sample volumes and thus new concepts for DACs. These new designs now enable high quality data obtained from DAC equipped with culet sizes of 700 and 800 µm to one megabar and above. Such neutron diffraction at megabar pressures can be expected to enable new science directions as well as answer pressing questions in geosciences, physics, chemistry and material science.

## Results

### Diamond cell designs

**Figure 1:**
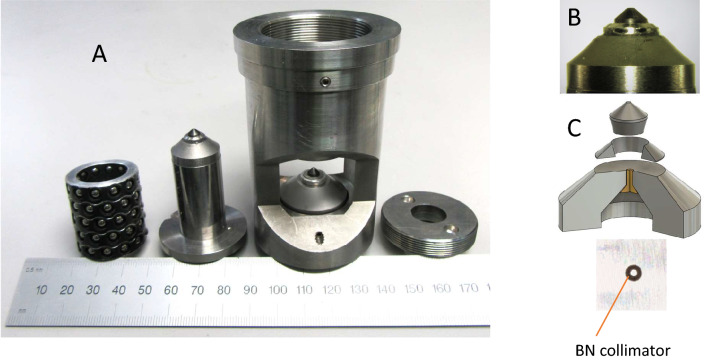
Megabar neutron diamond anvil cell. (**a**) A photo of the individual parts of the cell, roller bearings, top piston including seat and anvil, cell body including opposing seat and anvil, and top closing cap. (**b**) A photo of the seat and anvil including the steel binding ring used for additional support. (**c**) A 3D-schematic created in Autodesk Fusion 360 (V2.0.15050, https://www.autodesk.com/products/fusion-360) of seat and anvil including the collimator built into the seat consisting of amorphous boron with a small amount of epoxy. A close-up photo of the seat in top-view shows the aperture for incoming beam formed by the collimator. The diameter of the hole in the seat is thereby 1 mm diameter.

The diamond anvil cell for megabar neutron diffraction is an opposed-anvil piston-cylinder device with 80 degree aperture tailored for SNS’s dedicated high pressure neutron diffractometer SNAP^25,26^. The neutron beam enters along the pressure axis and scatters out radially from this pressure axis. Very high mechanical stability and accuracy in the cell body was achieved by using steel roller bearings between piston and cylinder shown in Fig. 1a. These roller bearings have essentially eliminated shear stresses that were always present in previous pressure cells, which were most likely the main cause for premature anvil failure. Further, these linear ball bearings have the additional advantage of nearly friction-less operation at low temperatures, eliminating the well-known ‘freezing’ of piston and cylinder in conventional cells.

New diamond anvil geometries were developed to accommodate optimal radial and axial support of the conical CVD diamond anvils. The anvils have dimensions of 5 mm in diameter and 4 mm height. The pavilion angle is 40$^{\circ }$ and the bottom cone angle is 30$^{\circ }$. The design accommodates for both axial and radial support of the anvil. Whereas conical support alone caused anvil failure due to plastic deformation or cracking of the seat (tungsten carbide or polycrystalline diamond)^23,24^, simple axial/flat support lead to splitting of the large diamonds along the axis due to elastic or plastic deformation of the seat material (steel or tungsten carbide). The combination of radial steel binding rings and high strength flat supports (tungsten carbide or steel) has significantly improved the stability of the anvils allowing routine experiments at over 50 GPa. Indeed, anvils with dimensions 5 $\times$ 4 mm could withstand forces up to 8 metric tons, or about 80 kN, without failure. Figure 1b,c show anvil and support combining axial and radial support. Culet sizes range from typically 600–900 µm allowing for sample volumes between 0.004 and 0.014 mm$^{3}$.

The seats of the anvils were made from tungsten carbide with an aperture of 23$^{\circ }$ (see Fig. 1). Since the incoming neutron beam is highly divergent and larger than the sample, the upstream seat requires an in-built collimator that allows for the absorption of all neutrons not illuminating the sample. This collimator is made from a mixture of boron powder (amorphous natural B or isotopically enriched crystalline $^{10}$B) and a small amount of epoxy. It is custom drilled for each experiment and allows for low background levels routinely adapted to a given culet size. Following its fabrication, the diamond anvil is optically aligned to the collimator. Typically, dimensions of culet, gasket hole and collimator are 700 µm, 300 µm and 280 µm, respectively. The alignment and effectiveness of the collimators are checked by monitoring diffraction peaks from the rhenium gasket described below.

The initially used stainless steel gasket (301, full hard)^23,24^ limited pressures to about 65 GPa. While Re, and to a lesser extent also W, have a high absorption cross-section for neutrons, this is not an issue for operation on SNS’s SNAP diffractometer. There, the large angular range available through the movable detectors allows for detector placements such that only small slivers of $2\theta$-range are attenuated by the gasket. Due to the use of TOF neutron diffraction rather than a monochromatic beam, this still allows for access to the full desired *Q*-range.

The megabar neutron DACs described here thus allow for the necessary high precision in alignment and operation, for the necessary stability in radial anvil support and gasket use as well as for sufficient sample volume as needed for high quality neutron diffraction data.

### Pressure-load curves

**Figure 2:**
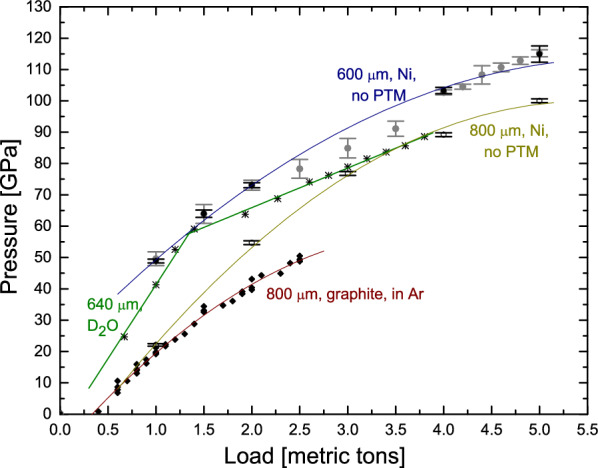
Pressure-load curves obtained from nickel, water ice and graphite. The pressure for the Ni and D$_2$O data were obtained from the diffraction data themselves using Ni and D$_2$O equations of state^30,31^, respectively. For those, pressures were determined through Gaussian fits to the diffraction data (black circles and stars, respectively). Additionally, for short runs, pressures were determined visually from the peak positions (grey circles) in one case. For the graphite loaded in Ar pressure was obtained using the ruby fluorescence method (black diamonds). Refer to the main text and the methods section for further details. In all cases, a fit was added to guide the eye. For Ni and graphite 2nd order polynomial fits were used, while two separate linear fits were to used to comply with a distinct change in slope at $\sim$ 60 GPa for ice.

A range of samples at various sample sizes and a maximum pressures have been run on the SNAP beamline using these anvil and cell designs. Here we show three different sample materials that cover a range of sample types: The first sample is solid nickel powder loaded without pressure medium, the second sample is water ice loaded as a liquid and the third sample is graphite loaded with gaseous argon used as pressure transmitting medium. In situ diffraction data were collected upon pressurization using a hydraulic DAC press^27^. The resulting pressure-load curves are shown in Fig. 2.

For the Ni powder, two different runs using 800 µm and 600 µm culets are shown. Pressure was determined from the accumulated data over the entire collection period and both banks. Following data reduction and background correction, the 111, 220 and 200 Ni reflections were separately fitted with a Gaussian each to extract the lattice parameter *a*, which was then used for pressure calculation. This was performed for all pressure points obtained with 800 µm culets and all long-exposure pressure points obtained with 600 µm culets. Additionally, pressure was determined visually from the data independently for the 111, 220 and 200 reflections for all data sets. Pressure was then calculated independently for each reflection and the pressure points shown on the pressure-load curve present the average while the uncertainty provided is the corresponding standard deviation. The actual variation due the positional/angular variations and or also pressure gradients may thus be higher. Further details are provided in the supplemental material.

For both Ni loadings a rapid, relatively linear pressure increase with load is seen initially, while the curve begins to flatten at higher loads indicating cupping of the anvil culets. The maximum pressure obtained with the 800 µm was 1 Mbar at 5 metric tons. Upon further load increase, the anvils collapsed at 5.13 metric tons. Specifically, $V/V_0$ = 0.7565 ± 0.0008 was measured experimentally. Using a Vinet EoS with $B_0$ = 183 GPa and $B'_0$ = 4.99^30^, this yields a pressure of 100 GPa. Note that all the Ni pressures presented in figures here are based on this EoS. However, it is noteworthy that a more recent EoS^32^ using a 3rd-order Birch Murnaghan with $B_0$ = 201 GPa, $B_0'$ = 4.4 yields a pressure of 104 GPa for this $V/V_0$. The cell with 600 µm culets achieved  1.15 Mbar at 5 metric tons. Specifically, $V/V_0$ = 0.738 ± 0.003 was measured experimentally. This equates to a pressure of 115 GPa using the Vinet EoS and of 120 GPa using the 3rd-order Birch–Murnaghan EoS quoted above. It should be noted that this cell failed after $\sim$ 1 h data collection at this load and pressure.

Next, a pressure-load curve obtained from water ice (here D$_2$O to enhance coherent neutron scattering) is shown. Unlike the solid Ni powder, this was a liquid sample loading. This presents additional challenges in terms of loading and gasket stability upon compression. As for the Ni, pressure was determined from the accumulated diffraction data, sampled over the entire exposure time and both detector areas. A Gaussian fit to the 110 reflection was used to extract the lattice parameter. To calculate pressure, we used $V_0$ = 12.3 cm$^3$ mol$^{-1}$, $B_0$ = 23.7 GPa and $B'_0$ = 4.15 with a Birch–Murnaghan finite-strain EoS^31^.

For this particular run on ice, the pressure was increased relatively rapidly up to 65 GPa, the pressure limit in most previous runs. Up to this pressure, short collection periods of 20 min were performed, which were sufficient to determine pressures from the main diffraction peaks and an equation of state. Above 65 GPa, longer data collections were performed. This yielded a maximum pressure of 88.6 GPa for the 640 µm culet used here. An intriguing kink is seen in the pressure-load curve at $\sim$ 60 GPa, i.e. in the pressure regime where previously catastrophic gasket/anvil-failures consistently occurred. Above and below the kink at $\sim$ 60 GPa, the pressure-load curve is linear, although at two different slopes. A further, more detailed offline experiment with H$_2$O loaded also in a neutron DAC but using ruby as a pressure calibrant, revealed no discontinuous slope change in the pressure load curve. Interestingly however, it did show that the pressure gradient across the sample chamber remained constant at 3–4 GPa upon pressure increase. A manuscript on the details of these findings is in preparation.

This gives useful insight into overall pressure gradients. While no significant broadening of diffraction lines was observed upon pressure increase, it should be noted that SNAP is a medium-resolution diffractomter only and thus broadening up to a certain point, may not be detected. Overall, this pressure gradient observed for H$_2$O is similar in magnitude to the variation/uncertainty in pressures determined for Ni, i.e. the variation due to different angular positioning ($\sim$ 6.5 GPa at 100 GPa, see supplemental). As in earlier X-ray diffraction studies, such pressure variation/gradients may in future be overcome using hydrostatic pressure media in combination with laser annealing.

The final pressure-load curve shown here is obtained from graphite measured (quasi-)hydrostatically with an Ar pressure transmitting medium loaded into the DAC at 2.6 kbar using ORNL’s gas loader^26^. Unlike the Ni and the ice examples, here the sample filled only a fraction the entire chamber to achieve (quasi-)hydrostatic conditions. This experiment demonstrates the capability of obtaining good diffraction patterns on even smaller samples, which is important for future experiments using for example metallic samples loaded in hydrogen.

Here, the pressure was obtained using the ruby fluorescence method. A hydrostatic ruby scale^30^ was used up to $\sim$ 20 GPa, at higher pressures the non-hydrostatic ruby scale^33^ was used instead. Pressure was measured before and after each data collection whereby pressure drifted upward by 1–3 GPa during long collections. All these measurements are included here. The graphite was loaded to 2.5 metric tons yielding a maximum pressure just above 50 GPa. At this pressure, the gasket started to deform asymmetrically and the cell was decompressed. The anvils were successfully recovered. This is noteworthy as compressed graphite has a tendency to break anvils.

### Data quality

**Figure 3:**
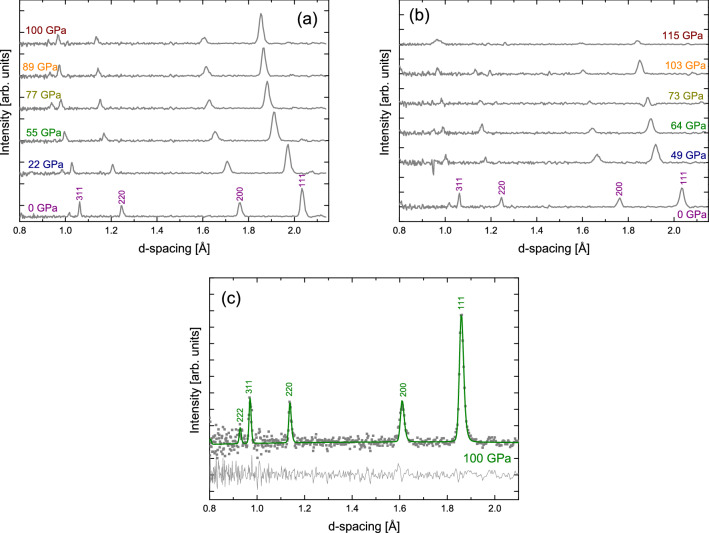
High pressure neutron diffraction of nickel. Neutron diffraction data obtained from Ni in a cell with (**a**) 800 µm culets and (**b**) 600 µm culets. (**c**) Quantitative Rietveld refinement performed on Ni measured at 100 GPa in the 800 µm culet cell after full background correction. The data set is presented by the measured data (grey), the calculated data (colored) and the difference function beneath. In all plots, Ni is additionally indexed as per Ni’s cubic unit cell, $Fm\overline{3} m$.

Despite the, in terms of neutron scattering, very small sample volumes, the data quality is sufficient for crystallographic analysis using conventional Rietveld approaches. While achieving megabar pressures for neutron diffraction is a critical breakthrough, it is of equally high importance that not only the positions of the Bragg peaks are determined accurately but also their intensities. This is critical pre-requisite to exploit the ability of neutron diffraction measurements to determine stoichiometry and atomic positions of light atoms such as in D$_2$O/H$_2$O ices, and metal superhydrides. An illustration of the data quality that can be achieved is demonstrated in Fig. 3.

Ni is chosen here as example element due to its high-symmetry cubic structure, its high coherent neutron scattering cross-section and the fact that it does not exhibit structure phase transitions in the pressure regime probed. The latter allows for reliable pressure determination from measured diffraction data as demonstrated above and benchmarks the accuracy of the data-reduction approach. Fig. 3a,b show the Ni diffraction data collected in DACs with 800 µm and 600 µm culets, respectively. The data were vanadium-normalized, to account for instrumental artefacts such as the incident flux wavelength dependence and detector efficiencies, (see details in supplemental material) and have had experimental backgrounds subtracted. Additionally, the data set shown in Fig. 3c have been corrected for attenuation of the incident beam by Bragg scattering of the upstream diamond attenuation^34^.

TOF diffraction is an energy-dispersive technique, whereby the transit time of detected neutrons can be used to resolve their energies. As a consequence, a full diffraction pattern can be extracted from neutrons scattered into even a small solid angle. When combined with large area detectors, this facilitates powerful insight into the sample microstructure, as observations at different angles sample the subsets of differently-oriented crystals relative to the beam (and, in our case, the load axis). Here, however, we have chosen to combine pixels from a wide angular range (spanning 61$^{\circ }$ to 126$^{\circ }$), as the increased total counts improve the statistics of our measurement. There are some considerations that must be made when combining data measured at different angles, which we discuss in the supplementary material.

In order to conduct the refinement shown, both the time-of-flight to *d*-spacing conversion and diffractometer resolution were determined from measurements of a calibrant powder of diamond collected with an identical angular range to the Ni data. This was done using standard calibration processes, developed for the GSAS-II^35^ Rietveld software package. The crystal structure of Ni has no refineable atomic positions, so the only crystallographic parameters refined were the lattice parameter and a single isotropic atomic-displacement parameter (ADP) for the Ni nucleus, $U_{iso}(Ni)$. The resulting fit, yielding a reduced-$\chi ^2=1.169$. The refined lattice parameter was $a = 3.2123(6)$Å and $U_{iso}(Ni)=0.0109 (27)$Å$^2$. Significant further improvement was found by adding a single-coefficient spherical-harmonic model of preferred orientation (PO) to the refinement. This dropped the reduced-$\chi ^2$ to 0.992 and visibly improved the fit, particularly in the vicinity of the 200 reflection and resulted in a texture index of 1.067. The lattice parameter was unchanged within error in the PO refinement, although the ADP did significantly reduce to $U_{iso}(Ni)=0.0030(27)$Å$^2$. This resulting fit is the one shown in Fig. 3c.

The good fit between a model of the well-known structure of Ni and our data demonstrates the reliability of our data-reduction procedures. Furthermore, this refinement quite clearly demonstrates that full, quantitative structural neutron refinements is achievable even at megabar pressures. In addition, the lack of sample-peak broadening, beyond the native instrumental resolution, suggests surprisingly low shear gradients, which are potentially being minimized by cupping of the (relatively large) diamond culets under our very high pressure conditions. Also notable is the low amount of PO, despite the absence of a pressure medium, which might be, at least partly, attributed to our ability to average over a large range of crystallite orientations for each Bragg peak we measure. Lastly, this angular range has the further advantage of delivering a good *Q*-range allowing for the detection of a good number of peaks even for high-symmetry systems such as Ni.

### Probing the high pressure behavior of graphite

Clearly these experiments on Ni demonstrate the achievement of megabar neutron diffraction together with the fact that full crystallographic analysis remains possible at these pressures. Next it is interesting to demonstrate that these new neutron DACs can indeed address challenging questions in high pressure science that are not readily studied by X-ray diffraction. These studies often require gas loading, involve low-Z materials and will typically probe materials with lower symmetry than cubic Ni or D$_2$O. Here, we demonstrate this on the example of graphite loaded in a DAC with Ar as soft pressure-transmitting medium. This is chosen for several reasons: (i) Carbon is a low-Z element and hence past X-ray diffraction DAC studies on graphite always have had to contend with high backgrounds arising from Compton-scattering of the diamond anvils. (ii) A study involving gas-loading highlights that data collection is possible even if the gasket chamber is not entirely filled with sample material. Furthermore, while gas-loading involved an inert gas here, the same procedure can be extended to hydrogen, for example. (iii) Graphite exhibits a hexagonal structure. (iv) Clearly the phase diagram of carbon continues to intrigue and many open questions remain.

Specifically here, the high pressure behavior of graphite remains of considerable interest as graphite does not transform directly to diamond upon compression. Instead intermediate phases form. A transparent phase forms between 14–18 GPa^36–38^, a formation that is critically dependent on the exact form of graphite used and on the compression conditions. These previous studies however, focused on optical methods, and thus the structure was not determined. While in situ synchrotron X-ray diffraction studies on graphite compressed within a DAC are challenging, some studies have been conducted. These indicate that this new structure is super-hard^39^, may possess the monoclinic structure of M-carbon but only nucleates very slowly over time^40^. The details of the nature of this structure, its dependence on the initial graphite used and the curious critical time-factor in nucleation still pose many open questions that could be addressed by high pressure neutron diffraction.

**Figure 4:**
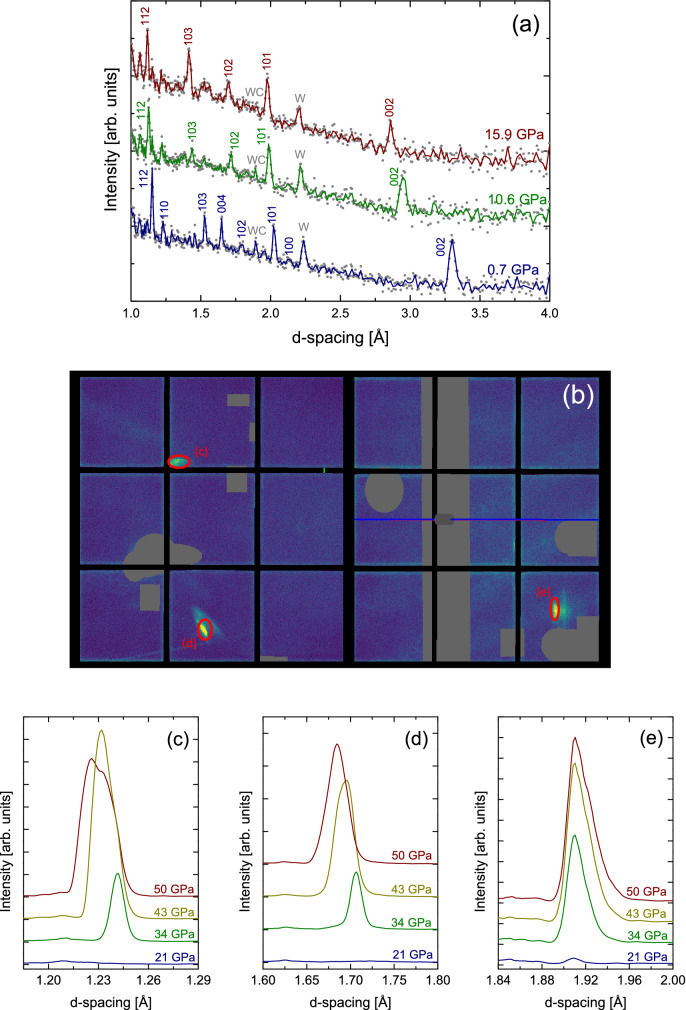
High pressure neutron diffraction of graphite loaded with Ar pressure medium. (**a**) Powder diffraction patterns from ambient pressure close to 16 GPa indexed based on graphite’s hexagonal unit cell, $P6_3/mmc$. Finely binned data are shown by (grey) circles and the solid lines show more coarsely binned data for clarity. (**b**) 2D detector view extracted from MantidWorkbench (V6.1.0, https://www.mantidproject.org/)^41^ showing the diffuse single crystal features observed at 50 GPa in a *d*-range of 1.1–2.1 Å. Cell backgrounds such as single crystal diamond peaks are masked (greyed out areas). The areas integrated for each individual single crystal feature are indicated by red circles. This was performed in the exact same detector location for each pressure from 21–50 GPa. The pressure evolution of the three peaks is shown in (**c**–**e**).

Here, diffraction data from graphite from ambient pressure to $\sim$ 50 GPa, are shown in Fig. 4. The initial pressure of 0.7 GPa was sealed in during gas loading. Interesting differences to past high pressure X-ray diffraction data^39,40^ are evident in this ambient pressure data set. Specifically, in our diffraction data, the 004 reflection is observed although it is typically absent in X-ray diffraction DAC data^39,40^. Closer inspection of our data resolved by angle rather than summarized over all detector banks shows that the 004 is not present in the same detector panels as the 002. Full crystallographic analysis is thus pending the development of multi-angle approaches to Rietveld refinement (see supplemental material).

As shown in Fig. 4a, upon compression to 10.6 GPa and 15.9 GPa, the typical large pressure-shift of the 002 and small pressure-shift of the majority of other reflections in observed. At the same time the 004 reflection was not readily observed any longer. Note that the pressures quoted are the starting pressure at data collection since pressure did drift up during collection, from an initial 8.7–10.6 GPa and from 14.3 to 15.9 GPa, respectively. Following the next pressure increase to $\sim$ 21 GPa, structural disordering occurred and the hexagonal graphite structure dissolved over time (not shown here). This may be consistent with past X-ray diffraction studies that see significant peak broadening at similar pressure^39^ or, following a wait time of 100+ hours, the appearance of new diffraction lines^40^.

Subsequent continued compression to $\sim$ 34 GPa resulted in the nucleation of a new crystal structure which persisted to the maximum pressure of $\sim$ 50 GPa probed here. Interestingly, this high pressure crystalline phase did not nucleate as random powder but as highly oriented, diffuse single crystal-like features. This is clearly seen in the 2D view of the two detector banks at the maximum pressure of 50 GPa (see Fig. 4b). Further pressure data sets and analysis details are available in the supplemental material. Integration of the three areas within the marked circles, i.e. of the diffuse single crystal features at various pressures, is shown in Fig. 4c,d. The peaks at $\sim$ 1.24 Å and $\sim$ 1.70 Å clearly shift with pressure, which confirms they are sample peaks. It is not clear if the peak at $\sim$ 1.91 Å shifts within the resolution-limit of SNAP, although very stiff behavior may be expected from a highly incompressible carbon phase. The number of peaks observed is insufficient for full structural analysis although the structure may yet be consistent with the previously observed M-carbon^40^. Further studies are needed to obtain a more complete set of diffraction peaks.

It is noteworthy that detection of these diffuse single-crystal features was most certainly aided by our use of white-beam TOF neutron diffraction, i.e. an energy-discriminating diffraction technique, coupled with large area detectors and the large angular scattering aperture of our DACs. The fact that an extremely oriented structure forms, may indeed contribute to some of the past difficulties in detecting high-pressure polymorphs of graphite in X-ray DACs. Full analysis of our findings, including multi-angular approaches, and additional experiments are underway and will be presented once completed. Nonetheless, it is clear that our neutron DACs can contribute new understanding to phase diagrams of light elements such as carbon.

### Discussion

Present developments in neutron DACs enable neutron diffraction above 1 Mbar for the first time. Remarkably, even at these high pressures data quality remains of sufficient quality for quantitative structure refinement, while retaining angular-resolved diffraction information on the sample. While previous breakout studies on ice have achieved pressures close to 1 Mbar^14,23^, the data quality in these studies was not of sufficient crystallographic quality to enable refinement and thus no scientific insight was gained. Yet, the possibility of refinement is a critical prerequisite for future works that require determination of detailed atomic positions and stoichiometry such as studies on metal hydrides, superhard nitrides, various ices, and many more. This capability for quantitative neutron diffraction at a megabar is developed within the context and framework of a neutron user facility. It is not only available to a few select researchers but is released and available to the wide community. This development thus opens many new possibilities and opportunities for a range of scientific areas and can aid in many pressing research questions that cannot readily be answered by synchrotron X-ray techniques.

Furthermore, it is useful to consider these pressure records and the correspondingly reducing sample volumes in the context of past developments for in situ X-ray powder diffraction in a DAC. The first in situ X-ray diffraction was conducted in 1977^42^ and 1 Mbar was achieved shortly after in 1978^1^. By the late 80s it was possible to perform multi-megabar experiments^43,44^. It took however, until the 2010s to perform experiments at maximum pressures of 6 Mbar^10–12^. In contrast, refinable neutron diffraction was only available up to $\sim$ 3 GPa until the early 90s. The development of the Paris-Edinburgh (PE) cell for in situ neutron diffraction presented a major breakthrough that allowed for fully refinable data at 10 GPa in 1992^45^ and $\sim$ 25 GPa in 1995^13^. The current record for refined data measured in the PE cell stands at 40 GPa achieved in 2019^14^. Higher pressures yet maintaining refinable data have only been achieved recently in neutron DACs allowing for pressures of 52 GPa in 2013^28^ and 62 GPa in 2019^29^. While other neutron DAC studies have also achieved pressures as high as 82 GPa^21^, data were not refined.

**Figure 5:**
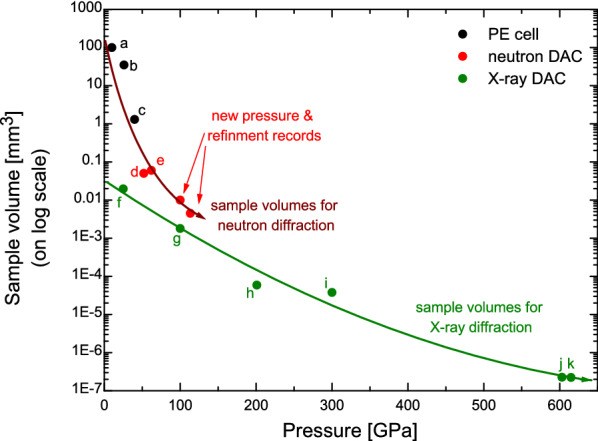
Sample volumes versus maximum pressure achieved during high pressure neutron and X-ray powder diffraction developments. For X-ray powder diffraction developments only DACs using gaskets are added here, for neutron powder diffraction developments, PE cells and neutron DACs (which are both using gaskets) are given. Lines indicating the volume reduction and increasing pressure records are added to guide the eye. Sample volumes during in situ high pressure powder diffraction development are taken from the literature as indicated by lower case letters: (**a**) first 10 GPa refined neutron powder data in a PE cell obtained in 1992^45^; (**b**) first PE cell pressure data at 26 GPa obtained in 1995^13^; (**c**) record 40 GPa pressure in the PE cell achieved in 2019^14^; (**d**,**e**) refined data obtained in a neutron DAC at 52 GPa and 62 GPa achieved in 2013 and 2019, respectively^28,29^; (**f**) the first in situ energy-dispersive X-ray powder diffraction performed up to 25 GPa in 1977^42^; (**g**) the first in situ X-ray diffraction measurements at 1 Mbar in 1978^1^; (**h**,**i**) very early 2 Mbar and 3 Mbar in situ X-ray diffraction performed in 1988^43^ and 1990^44^; and (**j**,**k**) representing the latest 6 Mbar and 6.15 Mbar records achieved in 2018^11,12^, respectively.

It is illuminating to compare the sample volume versus maximum pressure for X-ray and neutron powder diffraction as shown in Fig. 5. As is to be expected, the sample volumes required for high pressure neutron diffraction are considerably larger than those used for X-ray diffraction. For example, the first 25 GPa refinable data for neutron diffraction required close to 20 thousand times the sample volume of that used during the first synchrotron X-ray diffraction at similar pressures. The reduction in sample volume with increasing pressure has however, been significantly steeper for neutron diffraction. For synchrotron X-ray diffraction, the pressure increase from 25 GPa to 1 Mbar required a sample volume that was $\sim$ 10 times smaller. In contrast, the same pressure increase for neutron diffraction required a sample volume decrease by a factor of 3500. As a result, the sample volume we required for neutron powder diffraction at 1 Mbar is only $\sim$ 5–6 times the volume that had been used for the very first 1 Mbar synchrotron X-ray diffraction experiment.

Clearly, modern synchrotron and X-ray DAC techniques now enable significantly smaller sample volumes to be measured at 1 Mbar than these first experiments. Yet, neutron sources, neutron instrumentation and neutron DACs also continue to be developed. Advances are being made in overall neutron flux and brightness at new facilities such as the European Spallation Source or ORNL’s Second Target Station. Advances are made in instrumentation in terms of focusing guides and background reduction through collimation as well as the neutron DACs themselves. It is thus expected that sample volume requirements for neutron diffraction will decrease and will continue to approach those that are (or at least have previously been) used for synchrotron X-ray diffraction at multi-megabar pressures.

The megabar neutron DACs presented here are a major step toward opening neutron diffraction to similar pressures as routinely probed by synchrotron X-ray diffraction. By developing and releasing this capability, it becomes now possible to address science questions that require the complimentary information that can only be provided by neutron diffraction to a wide community.

## Methods

### Diamond cell preparation

Four different diamond cells were prepared as follows below. Cell 1 and cell 2 were loaded with Ni powder (<150 µm, 99.999% metal basis, Sigma-Aldrich), cell 3 was loaded with liquid D$_2$O (99.9 atom % D, Sigma-Aldrich) and cell 4 was loaded with graphite powder (graphite v598).

***Cell 1*** was prepared with tungsten carbide seats and an upstream collimator made from hexagonal boron nitride with a final hole drilled to 400 µm diameter. The cell was equipped with CVD anvils from Washington Diamond (WD Lab Grown Diamonds, Laurel, MD, USA) polished to a 800 µm culet without any bevelling. A Re gasket was indented to 80 µm and drilled to 400 µm diameter. The gasket was balanced on thin Al rings to avoid cupping. No pressure transmitting medium was used. The cell blew out during pressure increase at an applied load of 5.13 tons.

***Cell 2*** was prepared with tungsten carbide seats including an upstream collimator made natural amorphous boron powder mixed with epoxy with a final hole drilled to 250 µm diameter. The cell was equipped with CVD anvils from WD polished to a 600 µm culet with a double-bevel. A W gasket was indented to 70 µm and drilled to 300 µm diameter. No pressure transmitting medium was used. The cell blew out after holding an applied load of 5 tons for $\sim$ 15 min.

***Cell 3*** was prepared with tungsten carbide seats including an upstream collimator made from enriched boron powder ($^{10}$B) mixed with epoxy with a 2 mm long hole drilled to 250 µm diameter. The cell was equipped with CVD anvils from Almax (Almax easyLab, Diksmuide, Belgium) polished to a 640 µm culet with a 700 µm bevel. A W gasket was indented to 68 µm and drilled to 295 µm diameter. No pressure transmitting medium was used.

***Cell 4*** was prepared with tungsten carbide seats and an upstream collimator made from enriched boron powder ($^{10}$B) mixed with epoxy with a 3 mm long hole drilled to 340 µm diameter. The cell was equipped with 5.5 $\times$ 4.5 mm CVD from WD polished to a 800 µm culet with a 900 µm bevel. A W gasket was indented to 85 µm and drilled to 370 µm diameter. After loading sample and ruby balls for pressure measurement, the cell was gas loaded with Ar using the ORNL gas loader^26^. Upon an Ar gas pressure of 2.65 kbar, the cell was sealed inside the gas loader yielding a starting pressure of 0.7 GPa as per ruby fluorescence.

### Neutron diffraction

High pressure neutron diffraction was conducted at SNS’s dedicated time-of-flight high pressure diffractometer, the SNAP beamline^25^. Measurements were performed using its neutron focusing guide and a center wavelength of 2.1 Å at 60 Hz chopper speed. This yields a bandwidth of $\sim$ 0.5–3.65 Å. Detectors were placed at center positions of $2\theta = 65^{\circ }$ and $2\theta = 105^{\circ }$ for an optimized *Q*-coverage. The instrument was calibrated using a calibration standard to give correct lattice parameters, typically isotopic $^{11}$B-enriched NIST LaB$_6$ or standard diamond powder.

The diamond cells were placed into a hydraulic press for online compression^27^. Initial alignment of the cell on the beamline occurred via an alignment laser and via optics focused on the sample position for alignment along the beam. Alignment perpendicular to the beam was further refined using the neutron beam itself. Collection time at each pressure was as follows: 2.5 h for Ni in cell 1 (0.01 mm$^3$ sample volume); 5 h and 0.5–1 h for long and short runs for Ni in cell 2 (0.005 mm$^3$ sample volume), respectively; 0.33 h and 10–12 h for short and long runs for D$_2$O in cell 3 (0.0046 mm$^3$ sample volume), respectively; and 8–13 h for graphite in cell 4 (0.001 mm$^3$ starting gasket chamber volume).

### Data analysis

The resulting diffraction data were reduced using the Mantid framework^41^ employing the instrument calibration collected. As typical for all DAC data, the single-crystal diamond-anvil peaks were masked on the detector and removed from the powder data.

The Ni data were calibrated, normalized and background corrected. A select data set was further subjected to corrections arising from diamond attenuation^34^. In this case, subsequent Rietveld refinement was performed using GSAS-II^35^. For full details on the intricacies of the reduction and analysis procedure as necessary for quantitative crystallographic analysis, refer to the supplemental material.

The D$_2$O data were reduced using in Mantid the same methods as for our previous D$_2$O work^29^, namely calibration, vanadium normalization, absorption and background corrections, including those for diamond attenuation.

The graphite data were reduced in Mantid using basic SNAP routines. Normalization and background correction was performed using vanadium pin and empty instrument measurements. Additional backgrounds were removed via a fit to the remaining experimental background in Mantid and subtraction of this fit. The mantid masking tool was used to extract the single-crystal peak intensities at pressures above $\sim$ 30 GPa.

## Supplementary Information

Supplementary Information.

## Data Availability

All relevant data are available from the corresponding author upon reasonable request.

## References

[1] Mao HK, Bell PM, Shaner JW, Steinberg DJ (1978). Specific volume measurements of Cu, Mo, Pd, and Ag and calibration of the ruby R1 fluorescence pressure gauge from 0.06 to 1 Mbar. J. Appl. Phys..

[2] Boehler R (2020). High-pressure experiments and the phase diagram of lower mantle and core materials. Rev. Geophys..

[3] Silvera I (2020). The insulator-metal transition in hydrogen. Proc. Natl. Acad. Sci..

[4] Zerr A (1999). Synthesis of cubic silicone nitride. Nature.

[5] Boehler R (2005). Diamond cells and new materials. Mater. Today.

[6] Dubrovinsky L (2022). Materials synthesis at terapascal static pressures. Nature.

[7] Drozdov AP, Eremets MI, Troyan IA, Ksenofontov V, Shylin SI (2015). Conventional superconductivity at 203 Kelvin at high pressures in the sulfur hydride system. Nature.

[8] Somayazulu M (2019). Evidence for superconductivity above 260 K in lanthanum superhydride at megabar pressures. Phys. Rev. Lett..

[9] Kong P (2021). Superconductivity up to 243 K in the yttrium-hydrogen system under high pressure. Nat. Commun..

[10] Dubrovinsky L, Dubrovinskaia N, Prakapenka V, Abakumov A (2013). Implementation of micro-ball nanodiamond anvils for high-pressure studies above 6 Mbar. Nat. Commun..

[11] Dewaele A, Loubeyre P, Occelli F, Marie M, Mezouar O (2018). Toroidal diamond anvil cell for detailed measurements under extreme static pressures. Nat. Commun..

[12] Jenei Z (2018). Single crystal toroidal diamond anvils for high pressure experiments beyond 5 megabar. Nat. Commun..

[13] Klotz S (1995). Neutron powder diffraction at pressures beyond 25 GPa. Appl. Phys. Lett..

[14] Hattori T (2019). Development of a technique for high pressure neutron diffraction at 40 GPa with a Paris–Edinburgh press. High Press. Res..

[15] Klotz S, Casula M, Komatsu K, Machida S, Hattori T (2019). High-pressure structure and electronic properties of $${\rm ybd }_{2}$$ to 34 GPa. Phys. Rev. B.

[16] Goncharenko I, Loubeyre P (2005). Neutron and X-ray diffraction study of the broken symmetry phase transition in solid deuterium. Nature.

[17] Goncharenko I (2004). Neutron and X-ray diffraction study of the broken symmetry phase transition in solid deuterium. High Press. Res..

[18] Yamashita K (2020). A nano-polycrystalline diamond anvil cell with bulk metallic glass cylinder for single-crystal neutron diffraction. High Press. Res..

[19] Yamashita K (2022). Atomic distribution and local structure in ice VII from in situ neutron diffraction. Proc. Natl. Acad. Sci..

[20] Grzechnik A, Meven M, Friese K (2018). Single-crystal neutron diffraction in diamond anvil cells with hot neutrons. J. Appl. Crystallogr..

[21] Komatsu K (2020). Developments of nano-polycrystalline diamond anvil cells for neutron diffraction experiments. High Press. Res..

[22] Kozlenko D, Kichanov S, Lukin E, Savenko B (2018). The DN-6 neutron diffractometer for high-pressure research at half a megabar scale. Crystals.

[23] Boehler R (2013). Large-volume diamond cells for neutron diffraction above 90 GPa. High Press. Res..

[24] Boehler R, Molaison JJ, Haberl B (2017). Novel diamond cells for neutron diffraction using multi-carat CVD anvils. Rev. Sci. Instrum..

[25] Calder S (2018). A suite-level review of the neutron powder diffraction instruments at Oak Ridge National Laboratory. Rev. Sci. Instrum..

[26] Haberl B, Donnelly M-E, Molaison JJ, Guthrie M, Boehler R (2021). Methods for neutron diffraction studies on hydride superconductors and other metal hydrides. J. Appl. Phys..

[27] Boehler, R., Haberl, B., Molaison, J. & Guthrie, M. Development of large-volume diamond anvil cell for neutron diffraction: The neutron diamond anvil cell project at ORNL. In *Static and Dynamic High Pressure Mineral Physics* (eds Fei, Y. & Walter, M.) (Cambridge University Press, Cambridge, 2022). ISBN 978-1-108-47975-2.

[28] Guthrie M (2013). Neutron diffraction observations of interstitial protons in dense ice. Proc. Natl. Acad. Sci..

[29] Guthrie M (2019). Structure and disorder in ice VII on the approach to hydrogen-bond symmetrization. Phys. Rev. B.

[30] Dewaele A, Torrent M, Loubeyre P, Mezouar M (2008). Compression curves of transition metals in the mbar range: Experiments and projector augmented-wave calculations. Phys. Rev. B.

[31] Hemley R (1987). Static compression of H$$_2$$O-ice to 1.28 GPa (128 Mbar). Nature.

[32] Pigott J (2015). High-pressure, high-temperature equations of state using nanofabricated controlled-geometry Ni/SiO2/Ni double hot-plate samples. Geophys. Res. Lett..

[33] Mao HK, Xu J, Bell PM (1986). Calibration of the ruby pressure gauge to 800 kbar under quasi-hydrostatic conditions. J. Geophys. Res. Solid Earth.

[34] Guthrie M (2017). Radiation attenuation by single-crystal diamond windows. J. Appl. Crystallogr..

[35] Toby BH, Von Dreele RB (2013). GSAS-II: The genesis of a modern open-source all purpose crystallography software package. J. Appl. Crystallogr..

[36] Hanfland M, Syassen K, Sonnenschein R (1989). Optical reflectivity of graphite under pressure. Phys. Rev. B.

[37] Goncharov A, Makarenko I, Stishov S (1989). Graphite at pressures up to 55 GPa: Optical properties and Raman scattering. Phys. JETP.

[38] Utsumi W, Yagi T (1991). Light-transparent phase formed by room-temperature compression of graphite. Science.

[39] Mao W (2003). Bonding changes in compressed superhard graphite. Science.

[40] Wang Y, Panzik J, Kiefer B, Lee K (2011). Crystal structure of graphite under room-temperature compression and decompression. Sci. Rep..

[41] Arnold O (2014). Mantid—Data analysis and visualization package for neutron scattering and $$\mu $$SR experiments. Nucl. Instrum. Methods Phys. Res. Sect. A Accel. Spectrom. Detect. Assoc. Equip..

[42] Buras B, Olsen JS, Gerward L, Will G, Hinze E (1977). X-ray energy-dispersive diffractometry using synchrotron radiation. J. Appl. Crystallogr..

[43] Vohra YK, Duclos SJ, Brister KE, Ruoff AL (1988). Static pressure of 255 GPa (2.55 Mbar) by X-ray diffraction: comparison with extrapolation of the ruby pressure scale. Phys. Rev. Lett..

[44] Mao, H.K., Wu, Y., Chen, L.C., Shu, J.F. & Jephcoat, A.P. Static compression of iron to 300 GPa and Fe0.8Ni0.2 alloy to 260 GPa: Implications for composition of the core. J. Geophys. Res. Solid Earth **95**, 21737 (1990).

[45] Besson JM (1992). Equation of state of lithium deuteride from neutron diffraction under high pressure. Phys. Rev. B.

