# Supplementary material for: Advancing neutron diffraction for accurate structural measurement of light elements at megabar pressures
Source: Sci Rep. 2023 Mar 23;13:4741. doi: 10.1038/s41598-023-31295-3 (PMC10036630; doi:10.1038/s41598-023-31295-3)
Supplement: Supplementary file 1 — Supplementary Information. [file 41598_2023_31295_MOESM1_ESM.pdf]

Supplemental Material for 'Advancing neutron diffraction for accurate structural measurement of light elements at megabar pressures'

B. Haberl, M. Guthrie, R. Boehler

Neutron Scattering Division, Neutron Sciences Directorate, Oak Ridge National Laboratory, Oak Ridge, TN 37830, USA

1. Pressure determination

For the Ni samples, pressures were determined from the diffraction data rather than via independent methods such as ruby fluorescence. To confirm pressure readings and ensure accuracy, the lattice parameters used for pressure calculation were determined in two different ways:

(i) Through a Gaussian fit to the 111, 200 and 220 Ni peak whereby the data up were summed up over both detector banks. The pressure quoted is the average obtained from all three fits and the uncertainty provided is the standard deviation. This was chosen as it was somewhat larger than the uncertainty given based on the error propagated from the error yielded by the Gaussian.

(ii) Visually directly from the diffraction data whereby the data used were summed up over both detector banks. As above, the lattice parameter was determined independently from the 111, 220 and 200 Ni peaks. The pressure provided is the average pressure obtained from these three readings and the resulting uncertainty given is the standard deviation resulting from these same readings. This process was particularly useful for low-statistics data as those only collected for short times in the 600 μm culet case.

The pressures for the two runs performed with Ni, one with 800 μm culets and one with 600 μm culets are summarized in Tab. S1 and S2 below. These Pressures were calculated using a Vinet equation of state (B_0 = 183 GPa, B'_0 = 4.99) as provided by Dewaele *et al.* [1]. Note that a different more recent equation of state was also evaluated, as detailed in the main text.

The full complement of both methods was only possible for the data set obtained with 800 μm culets. Since the sample volume was significantly smaller for the data set obtained with 600 μm culets, significantly longer exposure times were needed for similar data quality. This was only possible for a few select pressure points. This complicated pressure analysis using fitting methods and thus, only a few select pressure points were analyzed through Gauss fitting. These are however sufficient to show very good agreement between both methods and thus confirm pressure determination.

Further, through the course of the data analysis, we noticed an angle-dependence of the pressure as observable upon angular inspection of the data. At the maximum pressure for the 800 μm culets this yields a variation of ~6.5 GPa from one edge of the 2θ-range to the other edge (with spread of 0.5 GPa only at ambient conditions). For the present work, only average pressures are presented, but future work will develop multi-angle approaches to investigate these behaviors in more depth.

Finally, no pressure transmitting medium was used for these Ni experiments. This may be expected to result in deviatoric stresses and shear gradients at megabar pressures. Unlike small culets used in equivalent X-ray diffraction experiments, it appears however that the large culets used here give rise to very significant cupping. This appears to allow for a more hydrostatic environment than seen with those smaller culets. The exact extant of the deviatoric stresses and shear gradients is thus not clear but presumed to be smaller than the angular variation mentioned above.

**Load [metric tons]**	**P from Gauss fit [GPa]**	**P read off data [GPa]**
0	0.0 ± 0.0	0.0
1	22.1 ± 0.4	21.8 ± 0.4
2	54.7 ± 0.6	54.3 ± 0.7
3	76.8 ± 0.6	76.8 ± 0.8
4	89.2 ± 0.6	89.6 ± 0.9
5	100.0 ± 0.6	99.5 ± 0.9

Table S1: *Pressures determined for Ni compressed in the DAC equipped with 800 μm culets. Note that the pressure quoted is the average obtained from three diffraction peaks (111,200 and 220) and the uncertainty presented in the table is the corresponding standard deviation. Note that V_0 was calculated in all cases using our own experimental lattice parameter a_0.*

**Load [metric tons]**	**P from Gauss fit [GPa]**	**P read off data [GPa]**
0	0.0 ± 1.2	0.0
1	48.9 ± 0.6	49.6 ± 2.2
1.5	64.0 ± 1.2	63.9 ± 3.0
2	73.1 ± 0.8	73.1 ± 1.6
2.5		78.3 ± 3.0
3		84.9 ± 3.1
3.5		91.1 ± 2.4
4	103.3 ± 1.0	102.7 ± 0.8
4.2		104.5 ± 0.8
4.4		108.3 ± 2.9
4.6		110.7 ±1.4
4.8		112.8 ±1.2
5	114.9 ± 2.6	115.2 ± 1.1

Table S2: *Pressures determined for Ni compressed in the DAC equipped with 600 μm culets. Note that the pressure quoted is the average obtained from three diffraction peaks (111,200 and 220) and the uncertainty presented in the table is the corresponding standard deviation. Note that V_0 was calculated in all cases using our own experimental lattice parameter a_0.*

2. Integration of single crystal graphite data

The graphite sample compressed to 50 GPa underwent a structural phase transition. Initial indication of the transition was the disappearance (or pseudomelting/amorpization) of the graphite, i.e. the Bragg peaks diminished in intensity over a time of 13 h at 21 GPa. This was followed by

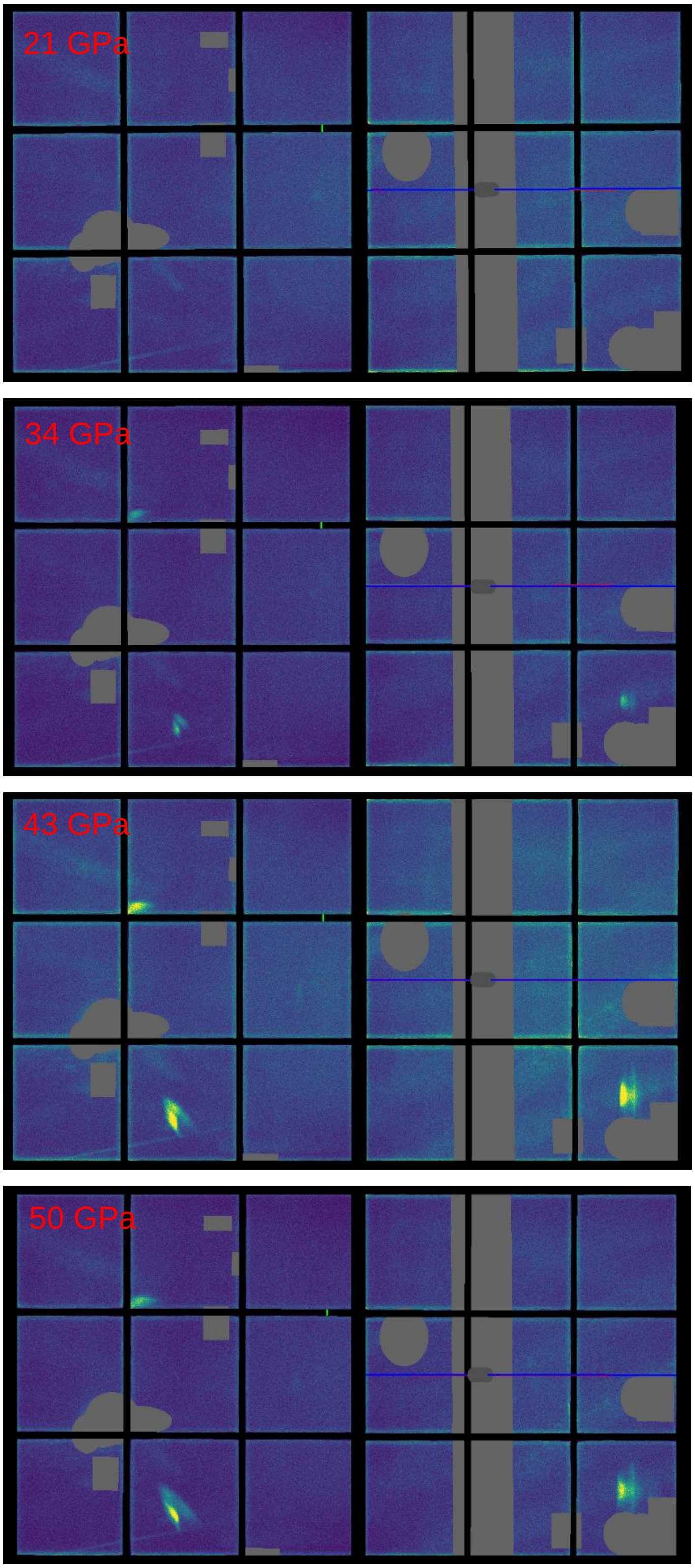

Figure S1: *2D detector view extracted from MantidWorkbench (V6.1.0, https://www.mantidproject.org/) [2] showing the two SNAP detector banks whereby each bank is made up of 3x3 modules upon increasing pressure. The d-range shown is 1.1-2.1 Å and the same rendering is used for all data sets.*

the nucleation of a highly ordered/oriented phase first observed at 34 GPa. Instead of a powder average that would be detected across the detector banks, single-crystal like peaks in three distinct locations were observed. Further compression increased the intensity of these observed single-crystal like peaks. The evolution of these peaks on the 2D detector banks is shown in Fig. S1. Note that masking (greyed out areas) was performed at 21 GPa, prior to formation of this new phase. This ensured that all background features were masked, i.e. single crystal diffraction arising from the single-crystal diamond anvils as well as some parasitic cell scatter arising from imperfect collimation within the cell itself, that was only noted after the experiment here.

For best analysis of these single crystal features and removal of all background, further masking was employed. Specifically, the entire module containing the single crystal feature was masked with exception of the feature itself, as shown in Fig. S2. Reduction software on SNAP allows routinely for reduction by module, which was then used following this masking. The 1D diffraction data shows in the main text are thus exclusively from these single crystal features and do not include any other detector area.

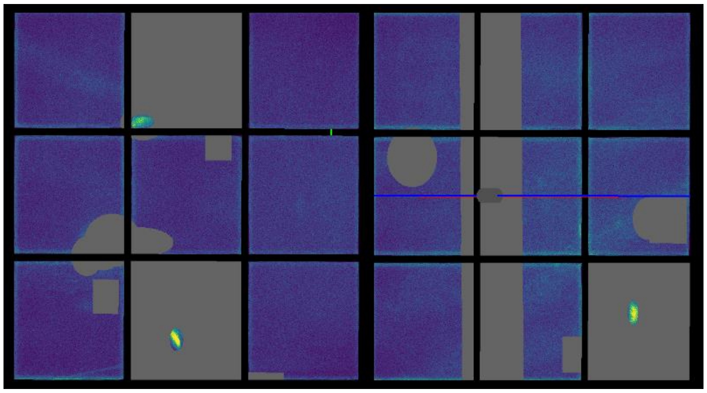

Figure S2: *2D detector view extracted from Mantid [2] showing the two SNAP detector banks at 50 GPa. The masking of the entire module with exception of the peak area is shown here.*

3. Neutron powder diffraction data reduction

The SNAP instrument has two identical square banks of detector arrays. Both banks are vertically centered on the equatorial plane of the instrument facing the sample, such that the detector faces are perpendicular to the line joining the sample to its center at a distance of ~500 mm. One bank is situated on the left-hand side of the beam and one on the right, and each has a rotation mechanism that allows them to be independently orientated at specific 2θ scattering angles within the equatorial plane. The total angular extent of each detector is 45° × 45° and, for all the measurements described here, the detector on the right-hand side of the beam was centered at nominal 2θ = 65 and that on the left-hand side centered at nominal 2θ = 105. Thus, by combining the total angular coverage of the detectors, a full range of $41 \leq 2\theta \leq 129$ scattering angles is sampled.

Each detector banks contains 9 separate modules arranged on a 3×3 square grid, and each module is a square grid of 256×256 individual pixels, yielding a total pixel count of 1179648. The raw experimental data consist of the detection of individual neutrons counts, called events, that are labelled with a unique pixel ID and the (absolute) time of arrival and time-of-flight (TOF) of the neutron. The TOF is the total transit time of the neutron from moderator to detector and can be readily measured as the neutrons are produced in sharp temporal pulses. As the neutrons are non-relativistic, TOF has a simple (parabolic) relation to the neutron wavelength and, therefore *d*-spacing, which can be calibrated using measurements of a known diffraction standard. By using this method, each individual pixel can detect a complete diffraction pattern arising from the subset of crystallites with their Bragg planes aligned perpendicular to the scattering vector for corresponding to that pixel.

Pixel grouping

The process of reducing the detected neutron events into meaningful diffraction intensities requires a series of steps whereby adjacent pixels are grouped and their neutron events summed. This is necessary because the small spatial extent of each individual pixel means a low event rate and correspondingly poor statistical determination of diffraction intensities. For the rigorous data an analysis performed on the Ni data in this paper, three distinct grouping schema are used in the reduction. The first of these involves the definition of "super"-pixels, whereby the events in adjacent 8×8 array of pixels are simply combined simulating the effect of having larger pixels. Although this lowers the intrinsic spatial resolution of the detectors, this is not significant, relative to the diffraction resolution of the instrument. Superpixel grouping is used in the calibration process described below.

A second grouping scheme is the combination of all events within the three vertical columns of modules in each detector, which each span a relatively large angular extent of approximately 15° horizontal and 45° vertical. The large angular extent of 'columnar' grouping would lead to unacceptable loss in diffraction resolution of the raw events were simply combined as they are in the much smaller superpixels. Correspondingly, it is necessary to transform the TOF of each neutron event to *d*-space *prior to summing* a process often referred to as *diffraction focusing*.

A third and final grouping scheme used in this work is to combine the individual columns into single spectra that effectively contain counts from the majority of pixels in the instrument. For reasons described below, this has been done in the final stage of the data reduction.

Geometric Calibration

The first step in the reduction process is to determine the relation between TOF and *d*-spacing. This was done using a measurement of a calibrant diamond powder (Sandvik Hyperion, SJK-5 grade, grain size between 0-0.5 μm), with a known lattice parameter of 3.567095 ± 0.000017 Å [3] and which was aligned at the precise center of the diffractometer via a combination of optical cameras and scanning through the neutron beam. The resultant diffraction signal was sufficient to

fit around 4-5 peaks in each superpixel using a simple Gaussian peak profile. The resultant ratios of peak positions, as a function of TOF, to known d-spacing of individual diamond diffraction peaks are averaged across all fitted peaks to give a linear diffraction constant (DIFC) for each super-pixel.

For all subsequent reduction processes, this constant conversion is used to transform between TOF and d-spacing on a superpixel-by-superpixel basis. Further, DIFC is proportional to the quantity $L \sin\theta$ (where L is the total path length from neutron moderator (the source) to the detecting pixel and θ the scattering angle). This derived from the De Broglie relation where $\lambda = \frac{h}{p} = \frac{h}{m_n v} = \left(\frac{h}{m_n}\right)\frac{T}{L}$ (with h = Planck, m_n = neutron mass, T = TOF, L = flight path) and Bragg's law $\lambda = 2d\sin\theta$. Substituting the De Broglie relation into Bragg's law yields: $\left(\frac{h}{m_n}\right)\frac{T}{L} = 2\sin\theta\, d,$ which can be converted into $T = 2\left(\frac{m_n}{h}\right) \mathrm{L}\sin\theta\, d.$ And thus $2\left(\frac{m_n}{h}\right)\mathrm{L}\sin\theta$ is the diffractometer constant for a given pixel. Since the neutron wavelength λ only depends on L it cannot be extracted from DIFC without knowledge of the angular location of a given superpixel. Correspondingly the transformation from TOF to λ is done in a different way, using the location of pixels as determined from metrological survey of the detector positions.

Wavelength normalization

The TOF diffraction technique is intrinsically an energy-dispersive diffraction approach. This introduces a need to normalize for the several wavelength-dependent factors that affect the detection of neutron events. These wavelength dependent factors include variation of the incident neutron flux profile, variation of detector efficiency, variation of guide efficiency and attenuation by various vacuum windows that lie in the upstream beam path. A standard approach is to measure the signal from a sample of pure vanadium metal. Vanadium has the uncommon property that almost all of the neutrons it scatters lose their phase and, therefore, do not combine to form coherent Bragg features. Instead, this scattering is taken to be wavelength-independent and so its measurement provides a determination of the multiplicative effect of all present wavelength-dependent factors, which can then be used to normalize the sample diffraction data.

This vanadium correction function is extracted from two experimental measurements: one measuring the scattering of a cylindrical rod of pure vanadium (600 μm diameter and several mm tall, which is significantly taller than the incident beam) and one measuring the intrinsic background signal of the empty instrument. In a first step, the focused background is subtracted to give the scattering signal of the vanadium alone, retaining information for individual superpixels. Subsequently, the events are converted to wavelength space and corrected for the vanadium self-absorption using a cylindrical correction that is applied for each pixel using the algorithm `CylinderAbsorption` provided in the mantid package [2]. The events are then transformed into d-space.

Subsequent steps in creating the vanadium correction are done following a columnar grouping of diffraction-focused pixels as described above and following an identical process to that used for the sample data.

Reduction of sample data in Columnar groups

The measured diffraction pattern has a strong dependence on both wavelength and scattering angle. The most dramatic angular effects are the shift of the fixed incident wavelength band to different d-spacing range and the degradation of diffraction resolution at lower angles. The former of these effects is readily inferred from Bragg's law $\lambda = 2d \sin \theta$ and the latter from its derivative with respect to θ. Additional angular effects appear from the Lorentz correction, which models angular variation in of the process whereby the Bragg condition is satisfied and, of course, geometric variation in the attenuation of the pressure cell itself.

Combining the data into columnar pixel groups, preceded by diffraction focusing, creates a tractable approach to analysis whereby each column can be assigned unique *d-range* and *d* binning parameters appropriate for the average diffraction resolution in the column without too severe degradation of resolution. Similarly, angle-dependent corrections can be applied also without strong negative effects from averaging across angles. Correspondingly, the workflow to reduce neutron events to diffraction intensities proceeds separately for each columnar group.

The first step in the workflow occurs during diffraction focusing. At this point a pixel mask, is used to remove any pixels affected by the strong background Bragg peaks from the diamond anvils. An important detail is that an identical mask is maintained throughout the workflow and this is also applied to the vanadium and attenuation corrections, which are thus diffraction focused in an identical way to the sample.

After masking and diffraction focusing of both the sample and vanadium correction function into (masked) columns, yielding functions in d-space, the vanadium correction is applied by division. The experimental background is then estimated using an algorithm that fits intensities outside the known location of sample peaks interpolating within these locations and then subtracted.

As a final step, the attenuation of the upstream diamond [4], a function of wavelength, is also converted to d-Space and diffraction focused taking account of the applied pixel mask. The resultant attenuation correction is applied by division.

The output of the above processes yields 6 separate, diffraction datasets that arise from separate orientational subsets of crystallites within the sample. A final effect to consider, is attenuation both by the sample itself and diamond-anvil cell surrounding it. The former effect can be estimated to be negligible as a consequence of small pathlengths, maximally 0.4 mm, and the relatively low absorption of nickel (4.49 barn for 2200 ms^{-1} neutrons). The primary contributor to the latter effect is the gasket material (rhenium and tungsten here), which absorbs strongly. The geometry of the gasket is such that this attenuation is strongly angle dependent and maximally effects the relatively small number of neutrons that travel directly through the gasket plane, a pathlength of several mm.

At the present moment, we do not have software to apply this attenuation correction, but this can be managed, to a certain extent, during refinement.

Combination of columnar groups and Rietveld analysis

For the Rietveld refinement shown in the main paper, we decided to merge all of the individual columnar datasets into a single spectrum. This has the advantage of maximizing the net statistics, but the disadvantage of compromising the higher experimental resolution of the high-scattering-angle columns. Furthermore, 'stitching' together the datasets introduced glitches coinciding with edges of individual column datasets and so we limited our range of refinement to the range of d-spacing common to all columns, this reduced the total d-range, quite significantly, but was sufficient to capture the longest d-reflection of nickel. A further concern was that the Lorentz correction, which has a $\sin\theta$ dependence, is not being fully applied while merging the data. However, the success of the resultant refinement, as described in the main manuscript, suggests that this does not have a significant effect. That might be attributed to $\sin\theta$ being closely linear within our measured range of angles, so the correction may reduce to a simple d-independent scale factor that is accounted for during refinement.

Another consideration is that we do not currently have the capability to calculate an attenuation correction for the gasket. To counteract this, we took the step of removing the most affected column (close to 90°). The remaining columns showed only minimal gasket attenuation. Lastly, we also observed that the lowest angle column had a very weak sample signal, likely due to shadowing by diffracted beam collimation and this too was removed. The final, summed spectrum, thus represents 4 of the 6 columns and 2/3 of the available pixels and spans a total range of scattering angle of $57 \leq 2\theta \leq 89$ and $97 \leq 2\theta \leq 129$ staggered about right and left of the instrument.

An important consequence of combining the columnar data into a merged spectrum is that we are also merging the diffraction signal arising from a wide distribution of crystallite orientation. This has an advantage, relative to monochromatic techniques of achieving better powder averaging. There is a possibility of anisotropic and or strain conditions within the sample and, again, our refinements sample the average of these.

Prior to refinement, the calibrant diamond sample was processed identically to the sample (minus the background subtraction and diamond-anvil cell attenuation corrections). This dataset is converted back to TOF, using a nominal diffractometer constant DIFC, which was then used to calibrate the resolution function of the combined columnar group. At the same time, the position of the diamond peaks was used to refine the TOF to d-spacing conversions, this time using a full three-term parabolic representation of the relationship (adding two additional diffractometer constants). This Rietveld calibration employed the GSAS-II software suite [5], which also allows the saving of the resultant instrument parameters to file. The nickel diffraction data, corresponding to the identical combined grouping of columns are converted to TOF using the same nominal DIFC and then read into GSAS-II, using the diamond instrument parameter file. This process ensures perfect mapping of the sample data into d-space.

The final refinements, described in the main paper, represent measurements of the unit cell of nickel averaged of the wide orientational distribution of crystallites sampled by our detectors. The surprising sharpness of the Bragg peaks, relative to instrumental resolution as measured by our diamond powder calibrant, indicates that within this angular range, there are relatively low strain distributions. Further analysis conducted on *individual angular banks,* in principle, allows extraction of the angular pressure distributions. It also will allow direct measurement of preferred orientation, although the relative intensities of the averaged sample already suggest that this is minimal. Although extracting these details is beyond the scope of the present manuscript, we are actively developing full multi-angle approaches to Rietveld refinement of our datasets that will enable such studies.

References:

[1] Dewaele, A., Torrent, M., Loubeyre, P. & Mezouar, M. Compression curves of transition metals in the mbar range: Experiments and projector augmented-wave calculations. *Phys. Rev. B* **78**, 104102 (2008).

[2] Arnold, O. *et al.* Mantid - Data analysis and visualization package for neutron scattering and μSR experiments. *Nuclear Instruments and Methods in Physics Research Section A: Accelerators, Spectrometers, Detectors and Associated Equipment* **764**, 156–166 (2014).

[3] Shikata, S., Tanno, T., Teraji, T., Kanda, H., Yamada, T. & Kushibiki H.-i., Precise measurements of diamond lattice constant using Bond method, *Japanese Journal of Applied Physics* **57**, 111301 (2018).

[4] Guthrie, M. *et al.* Radiation attenuation by single-crystal diamond windows. *Journal of Applied Crystallography* **50**, 76–86 (2017).

[5] Toby, B. H. & Von Dreele, R. B. GSAS-II: the genesis of a modern open-source all purpose crystallography software package. *Journal of Applied Crystallography* **46**, 544–549 (2013).
